# Bile acid supplementation decreases body mass gain in C57BL/6J but not 129S6/SvEvTac mice without increasing energy expenditure

**DOI:** 10.1038/s41598-018-37464-z

**Published:** 2019-01-15

**Authors:** Tobias Fromme, Kristina Hüttinger, Stefanie Maurer, Yongguo Li, Thomas Gantert, Jarlei Fiamoncini, Hannelore Daniel, Sören Westphal, Martin Klingenspor

**Affiliations:** 1 Chair of Molecular Nutritional Medicine, TUM School of Life Sciences, Technical University of Munich, Freising, Germany; 2 EKFZ - Else Kröner-Fresenius Center for Nutritional Medicine, Technical University of Munich, Freising, Germany; 3 ZIEL - Institute for Food & Health, Technical University of Munich, Freising, Germany; 4 Molecular Nutrition Unit, Technical University of Munich, Freising, Germany; 5 Department of Internal Medicine II, University of Ulm, Ulm, Germany

## Abstract

Supplementation of cholate to a high fat diet can protect mice from diet-induced, increased body mass gain. It has been hypothesized that uncoupling protein 1 dependent, non-shivering thermogenesis in brown adipocytes provides the mechanism of increased energy expenditure to counteract excessive energy intake. We scrutinized this conjecture in wildtype mice and mice genetically devoid of a functional uncoupling protein 1 gene (C57BL/6J) as well as mice of the 129S6/SvEvTac strain that, in comparison, display an extraordinary capacity to recruit ectopic brown adipocytes. Protection from diet-induced, increased body mass gain by cholate supplementation was absent in 129S6/SvEvTac mice, a consequence of much lower bile acid absorption and spillover in this strain. Conversely, Ucp1-KO mice did not differ from C57BL/6J wildtype controls in any parameter assessed. Daily energy expenditure and resting metabolic rate of C57BL/6J mice remained unaffected by cholate supplementation. We conclude that protection of mice from diet-induced, increased body mass gain by cholate supplementation depends on the specific genetic background of C57BL/6J mice, does not involve increased energy expenditure and is independent of uncoupling protein 1 dependent non-shivering thermogenesis.

## Introduction

Dietary supplementation of bile acids protects mice from diet-induced, increased body mass gain^1–3^. Bile acids have long been recognized as endocrine regulators beyond their role as emulsifiers in the gut lumen. Most prominently, bile acids act as steroid hormones by binding to the farnesoid X receptor (FXR) in hepatocytes to feedback-regulate their own synthesis (reviewed in^4^). To exert endocrine effects, bile acids can escape enterohepatic circulation by spillover from the portal into the systemic bloodstream. Peripheral tissues sense bile acids either by FXR or by the G-protein coupled bile acid receptor (GPBAR1, formerly TGR5), a signaling pathway associated with increased energy expenditure caused either directly or secondarily by thyroid hormone activation^1,5,6^.

The amount of systemically circulating bile acids can be modified by dietary supplementation of bile acids or by pharmacological agents interfering in bile acid homeostasis. In such intervention studies, changes in plasma bile acid levels positively correlated with concomitant changes in energy expenditure^1,7,8^. The underlying mechanism has been proposed to rely on binding of circulating bile acids to GPBAR1 on the surface of brown adipocytes to activate non-shivering thermogenesis^1–3^. This brown adipocyte-specific, powerful thermogenic process requires the presence of uncoupling protein 1 (UCP1) and would well be able to provide sufficient capacity to explain all changes in energy expenditure observed in response to altering bile acid levels (reviewed in^9^).

UCP1-dependent, non-shivering thermogenesis does not only occur within brown adipose tissue, but also in so-called brite (brown in white) adipocytes found interspersed within white fat depots^10,11^. Intriguingly, dietary bile acid supplementation leads to an increased abundance of brite cells^2,3^, probably by interaction with GPBAR1^12^. In the present study, we explored the contribution of brown and brite adipocytes to bile acid-mediated changes in energy expenditure and body mass gain in cell culture and in three mouse models. We employed wildtype mice and mice genetically devoid of a functional Ucp1 gene (C57BL/6J) as well as mice of the 129S6/SvEvTac strain that, in comparison, display an extraordinary capacity to recruit brite adipocytes^13^.

## Results

### Protection from diet-induced, increased body mass gain by cholate supplementation is mouse strain specific

Supplementation of cholate to a high fat diet has been reported to protect mice from diet-induced, increased body mass gain^1^. It has been hypothesized that uncoupling protein 1 (UCP1)-dependent non-shivering thermogenesis in brown adipose tissue (BAT) provides the mechanism of increased energy expenditure to counteract excessive energy intake^1–3^. Apart from BAT, thermogenically competent brown-like adipocytes reside interspersed in white adipose tissue (WAT) depots (brite/beige adipocytes)^10,14,15^.

**Figure 1:**
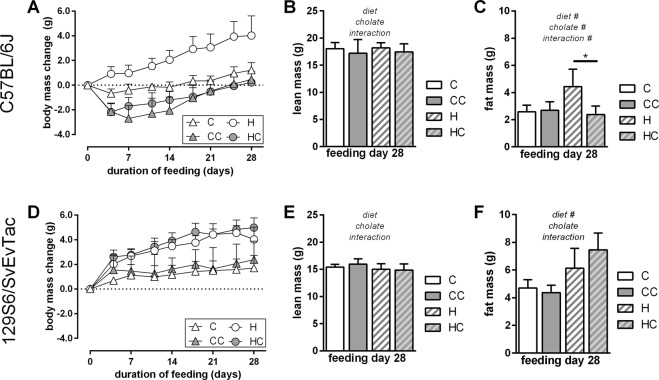
Dietary bile acid supplementation in two mouse strains, C57BL/6J (panels A–C) and 129S6/SvEvTac (panels D–F), fed control or high fat diet (C and H, white fill) and the respective versions supplemented with cholate (CC and HC, grey fill). (**A**) Body mass change trajectories along four weeks of feeding trial. Cholate supplementation decreased body mass accumulation in both diet groups with a stronger effect in high fat diet (LME). (**B**) Lean mass after completion of the feeding trial. (**C**) Fat mass after completion of the feeding trial. (**D**) Body mass change trajectories along four weeks of feeding trial. Cholate supplementation had no effect in any group of 129S6/SvEvTac mice (LME). (**E**) Lean mass after completion of the feeding trial. (**F**) Fat mass after completion of the feeding trial. All data are mean values ± SD; n = 6–7 (**A**–**C**) and 5–6 (**D**,**F**); results of 2-way-ANOVA in italics (**B**,**C**,**E**,**F**) with ^#^significant effect; *significantly different (t-test post-hoc); LME = tested by linear mixed effects model fit, see materials and methods.

Dietary bile acid supplementation leads to browning of WAT *in vivo*^2,3^. We here compared the effects of cholate supplementation in two strains of mice known to differ in number and recruitment capacity of brite adipocytes, C57BL/6J and 129S6/SvEvTac, to uncover a possible contribution of these cells to the effect of dietary bile acids^13^. In mice of the C57BL/6J strain, we also observed a strong protection from diet-induced body mass gain by dietary cholate supplementation (Fig. 1A–C). In both control and high fat diet fed mice, cholate led to a transient decrease in body mass followed by a sustained lower body mass (Fig. 1A). This difference in body mass was caused by a different fat mass and not by altered lean mass (Fig. 1B,C). These observations are in line with previous studies^1–3^.

Surprisingly, mice of the more browning-susceptible 129S6/SvEvTac strain proved to be fully resistant to any effect of bile acid supplementation (Fig. 1D,F). Both feeding a supplemented control and a high fat diet led to similar body mass trajectories and final body compositions as compared to the respective diet without cholate. Cholate did not at all protect 129S6/SvEvTac mice from high fat diet-induced, increased body mass gain. It thus acted in a mouse strain specific manner.

### Recruitment of brite adipocytes by cholate supplementation is mouse strain specific

**Figure 2:**
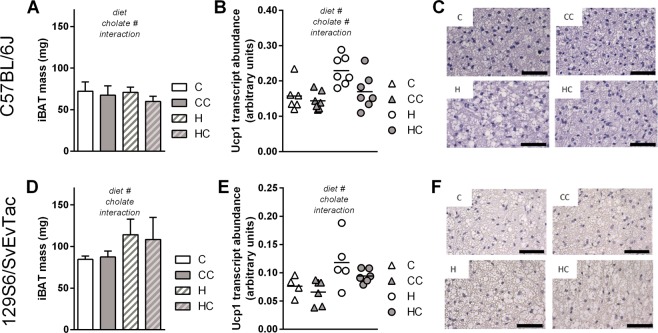
Dietary bile acid supplementation in two mouse strains, C57BL/6J (panels A–C) and 129S6/SvEvTac (panels D–F), with control (C) or high fat (H) diet and the respective versions supplemented with cholate (CC and HC). (**A**) Interscapular brown adipose tissue (iBAT) mass after the 4 weeks feeding trial, mean ± SD, n = 6–7. (**B**) Transcript abundance of uncoupling protein 1 (UCP1) in iBAT as shown in panel A, horizontal bar = mean. (**C**) Histological appearance of representative, hematoxylin-eosin-stained iBAT sections from the four diet groups, scale bar 50 µm. (**D**) Interscapular brown adipose tissue (iBAT) mass after the 4 weeks feeding trial, mean ± SD. (**E**) Transcript abundance of uncoupling protein 1 (UCP1) in iBAT as shown in panel D, horizontal bar = mean. (**F**) Histological appearance of representative, hematoxylin-eosin-stained iBAT sections from the four diet groups, scale bar 50 µm. All bars are mean values ± SD; n = 6–7 (A + B) and 5–6 (D + E); results of 2-way-ANOVA in italics (**A**,**B**,**D**,**E**) with ^#^significant effect.

In cholate sensitive C57BL/6J mice, BAT mass was slightly reduced by cholate supplementation (Fig. 2A) accompanied by a reduced lipid content as judged visually from histological sections (Fig. 2C). Transcript abundance of thermogenic UCP1, however, was reduced by cholate (Fig. 2B). Cholate resistant 129S6/SvEvTac mice again displayed none of these effects (Fig. 2D,F).

**Figure 3:**
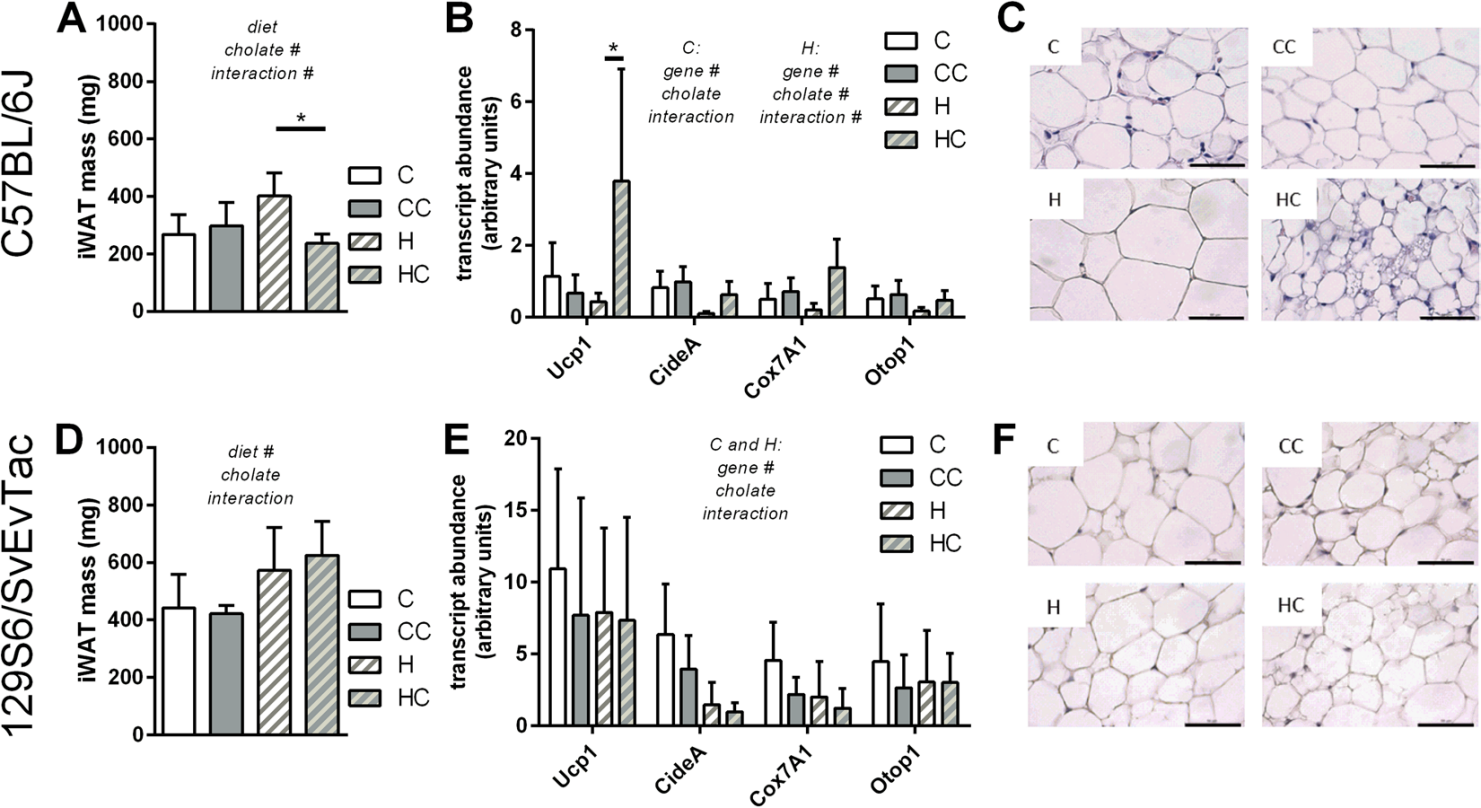
Dietary bile acid supplementation in two mouse strains, C57BL/6J (panels A–C) and 129S6/SvEvTac (panels D–F), with control (C) or high fat (H) diet and the respective versions supplemented with cholate (CC and HC). (**A**) Inguinal white adipose tissue (iWAT) mass after the 4 weeks feeding trial, mean ± SD. (**B**) Transcript abundance of uncoupling protein 1 (UCP1) in iBAT as shown in panel A, horizontal bar = mean. (**C**) Histological appearance of representative, hematoxylin-eosin-stained iWAT sections from the four diet groups, scale bar 50 µm. (**D**) Inguinal white adipose tissue (iWAT) mass after the 4 weeks feeding trial, mean ± SD. (**E**) Transcript abundance of uncoupling protein 1 (UCP1) in iWAT as shown in panel D, horizontal bar = mean. (**F**) Histological appearance of representative, hematoxylin-eosin-stained iWAT sections from the four diet groups, scale bar 50 µm. All bars are mean values ± SD; n = 6–7 (A + B) and 5–6 (D + E); results of 2-way-ANOVA in italics (**A**,**B**,**D**,**E**) with ^#^significant effect, *significantly different (Sidak’s multiple testing post-hoc).

Inguinal white adipose tissue (iWAT) is a depot with a considerable content of brite adipocytes and a high browning capacity, even more so in the 129S6/SvEvTac as compared to the C57BL/6J strain^13^. In C57BL/6J mice, iWAT mass increased in response to high fat diet feeding, but remained comparable in mass to control diet in the cholate-supplemented group (Fig. 3A). The depot thereby mirrors changes in total body fat mass (Fig. 1C). In iWAT, cholate led to a several fold increase in UCP1 transcript abundance when supplemented to a high fat diet, but not with control diet (Fig. 3B). For other markers of brite cell abundance (cell death-inducing DNA fragmentation factor alpha-like effector A, cytochrome C oxidase subunit 7a2, otopetrin 1), cholate supplementation rather alleviated high fat diet-induced reduction, a trend that failed to reach significance. The number of UCP1-positive, multilocular cells was clearly higher when feeding a high fat diet supplemented with cholate as compared to all other diets (Fig. 3C, Suppl. Fig. S1). These findings corroborate earlier reports on bile acid mediated WAT browning^2,3,12^. It remains to be clarified whether browning is a direct effect of circulating bile acids on (pre-)adipocytes or mediated by an intermediate signal. At least in our hands, cultured white preadipocytes failed to display brown adipocyte specific gene expression in response to bile acid treatment (Suppl. Fig. 2A). On the organismic level, it is questionable whether a four-fold increase of UCP1 transcript in iWAT compensates the reverse, significant reduction in transcript abundance in BAT taking into account very much higher absolute UCP1 amounts in the latter^16^.

We initially included 129S6/SvEvTac mice in this study for their high number of brite adipocytes and their susceptibility to browning stimuli. The accumulation of body fat mass during high fat diet feeding was independent of cholate (Fig. 1F) and was reflected on the level of iWAT depot mass (Fig. 3D). We indeed found a high amount of UCP1 and other brite marker transcripts in iWAT of these mice, but they were not altered by any diet or supplementation (Fig. 3E).

Taken together, we corroborate earlier findings on bile acid mediated protection from diet induced increased body mass gain in the C57BL/6J mouse strain and the accompanied recruitment of UCP1 in white, but not in brown adipose tissue. However, we did not observe any such effect in 129S6/SvEvTac mice that proved completely unresponsive to cholate feeding.

### Protection from diet-induced, increase body mass gain requires sufficient bile acid absorption

In contrast to mice of the C57BL/6J strain, 129S6/SvEvTac mice did not react to dietary cholate supplementation in any parameter measured in this study. To clarify whether this discrepancy is caused by differences in intestinal absorption, metabolism or tissue sensitivity, we determined bile acid concentration and composition in several organismic compartments.

**Figure 4:**
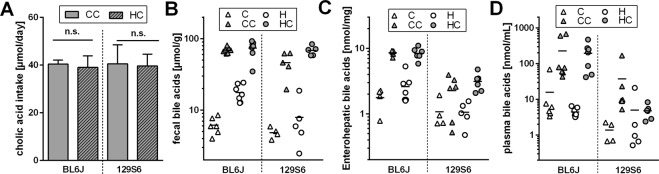
Bile acid intake and organismic distribution in mice of two strains, C57BL/6J and 129S6/SvEvTac fed control or high fat diet (C and H, white fill) or the respective versions supplemented with cholate (CC and HC, grey fill). (**A**) Mean daily cholate intake from supplemented diets during the 4 week feeding trial, mean ± SD, n.s. = not significantly different (t-test). (**B**) Fecal bile acid excretion is increased by cholate supplementaion in both diets (p < 0.0001). (**C**) Bile acid amount sequestered within enterohepatic organs is increased by cholate supplementaion in both diets (p < 0.0001, but much less so in 129S6/SvEvTac mice (p < 0.0001). (**D**) Plasma bile acid concentration is increased by cholate supplementaion to high fat diet (p < 0.05), but much less so in 129S6/SvEvTac mice (p < 0.05). Trends are similar in the control diet groups. Group comparisons in panels B–D were conducted by separate 2-way ANOVAs for C and H diets, n = 4–7.

Mice of both strains ingested similar amounts of cholate on both control and high fat diet (Fig. 4A). The total amount of bile acids in feces was drastically increased by cholate supplementation, but did not differ greatly between strains (Fig. 4B). In the complete enterohepatic organ, however, the amount of bile acids was strongly elevated by cholate supplementation in C57BL/6J, but much less so in 129S6/SvEvTac mice, which displayed only a third of bile acid amount in this organ system after supplementation (Fig. 4C). Thus, of a similar amount of ingested cholate, a much higher proportion was seemingly absorbed and recycled in C57BL/6J mice.

Additionally, mice of the 129S6/SvEvTac strain displayed a much lower plasma bile acid level than C57BL/6J, cumulating in a >30-fold difference after supplementation of cholate to a high fat diet (Fig. 4D). A trend to this difference already occurred in the absence of cholate supplementation and with both diets. We conclude that the spillover of bile acids from portal into systemic circulation is much more pronounced in C57BL/6J mice at all times, especially when challenged with a high dietary cholate intake.

Interestingly, the contribution of individual bile acids to the total pool was similar in both strains upon supplementation of cholate and only differed by compartment (feces, enterohepatic organ, plasma) as shown in Suppl. Fig. S3. Therefore, the lack of any metabolic consequence to cholate feeding in 129S6/SvEvTac mice was not a consequence of altered pool composition, but of total concentration.

Taken together, mice of the 129S6/SvEvTac strain absorbed a lower proportion of ingested bile acids with lower levels in enterohepatic circulation and displayed a lower spillover rate of bile acids into systemic circulation. This may be one causal aspect of the stark contrast in metabolic consequences of manipulating the microbiome in these strains^17,18^. Transcript abundance of key bile acid handling gene products was similar in liver and ileum of the two mouse strains (Suppl. Fig. S7). A further comparative, genetic dissection of the mouse strains C57BL/6J and 129S6/SvEvTac will be valuable to further elucidate the molecular and mechanistic determinants of these processes.

### Cholate supplementation does not increase energy expenditure

**Figure 5:**
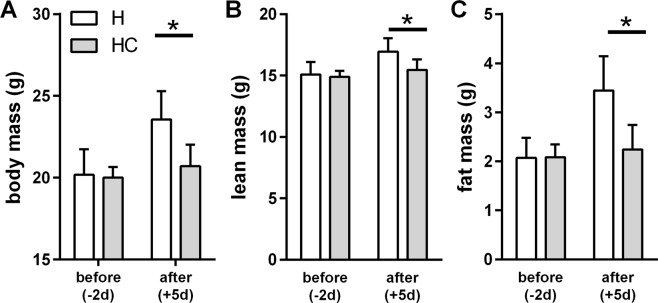
Body composition of C57BL/6J mice before (2 days before start of calorimetry and diet change) and after (5 days) of feeding trial with high fat diet supplemented with cholate (HC, grey fill) or not (H, white fill). (**A**) Body mass. (**B**) Lean mass. (**C**) Fat mass. All bars are mean values ± SD; n = 11; *significantly different (t-test on diet effect after 5 days).

Mice of the C57BL/6J strain were protected from high fat diet-induced body mass gain by cholate. The mass difference mainly manifested during the first week of supplementation and persisted thereafter (Fig. 1A). We therefore determined energy expenditure during one week, starting two days before mice were switched to high fat diet. Again, cholate supplementation protected mice from high fat diet-induced body mass and fat mass gain (Fig. 5A–C). After five days of high fat diet feeding, mice fed a diet supplemented with cholate weighed approximatively 2 g less than mice fed an unsupplemented diet.

**Figure 6:**
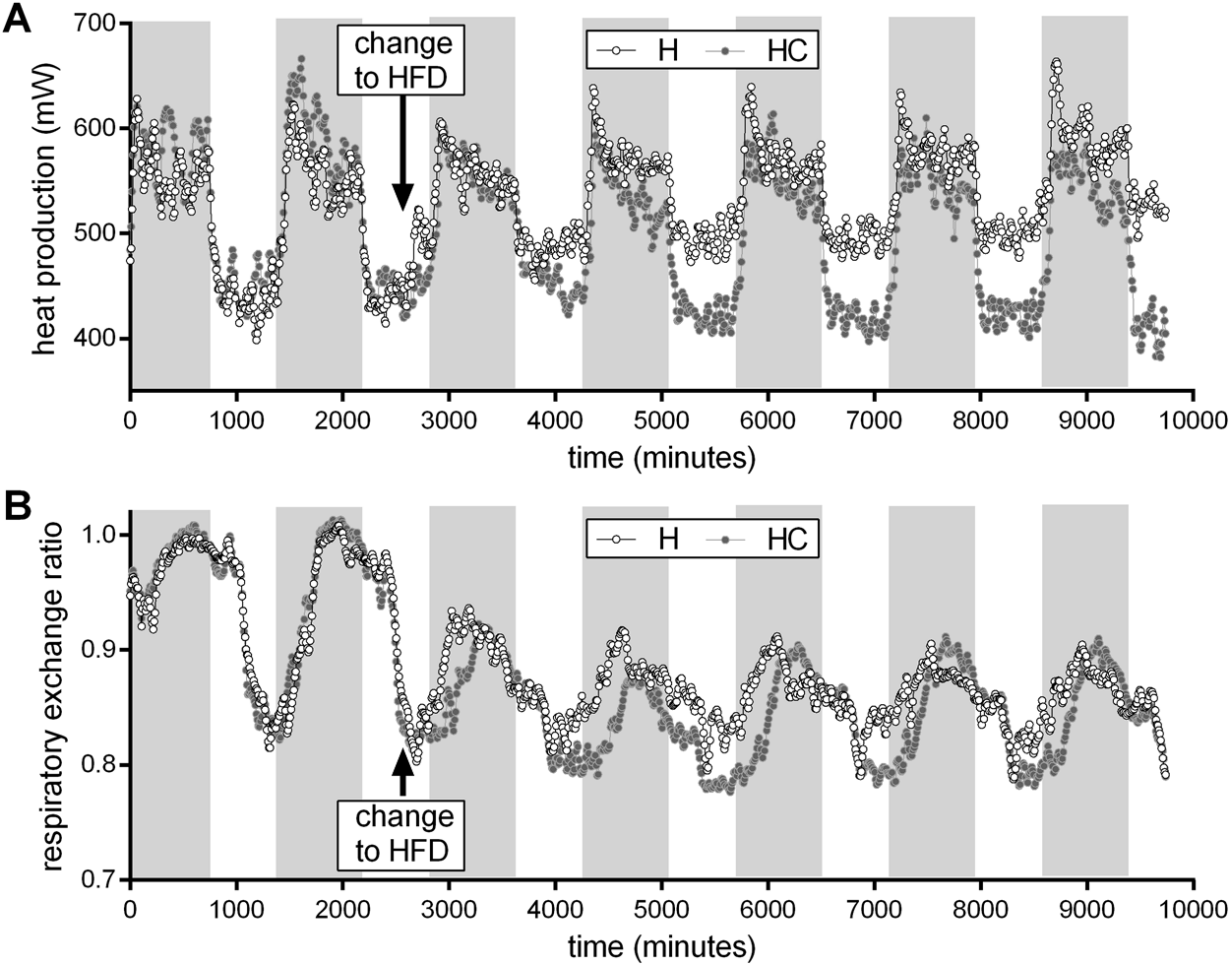
Indirect calorimetry of C57BL/6J mice during a feeding trial with high fat diet supplemented with cholate (HC, grey fill) or not (H, white fill). (**A**) Heat production of diet groups during two days before and five days after diet change. (**B**) Respiratory exchange ratio of diet groups during two days before and five days after diet change. Every symbol represents the mean of 11 individual mice. Alternating background shading illustrates the 12:12 h light-dark cycle.

We determined substrate use inferred from the respiratory exchange ratio (RER) and heat production of mice by continuous indirect calorimetry. All mice displayed the typical circadian rhythm of nocturnal activity accompanied by high heat production and preferred carbohydrate usage as compared to lower heat production and preferred lipid use during daytime (Fig. 6A,B). The diurnal pattern was principally preserved after the diet change in both groups. In accordance with a higher dietary fat content and thus a lower food quotient, RER decreased in all mice after the diet switch (Fig. 6B). This decrease was more pronounced when the diet was supplemented with cholate, especially during light- and early dark phase. Typically, food intake of mice arousing around light-off causes a rise in RER during the early dark phase. This increase was markedly delayed in the cholate-supplemented group suggesting a postponed onset of food intake. Differences in RER between the supplemented and the non-supplemented diet group appeared rapidly after diet change and were thus not secondary to changes in body mass and composition.

Starting with the switch to high fat diet, heat production successively increased, especially while at rest during daytime (Fig. 6A). This increase in heat production over time was absent in mice fed a high fat diet supplemented with cholate. In other words, we did not find evidence for increased energy expenditure caused by cholate supplementation.

**Figure 7:**
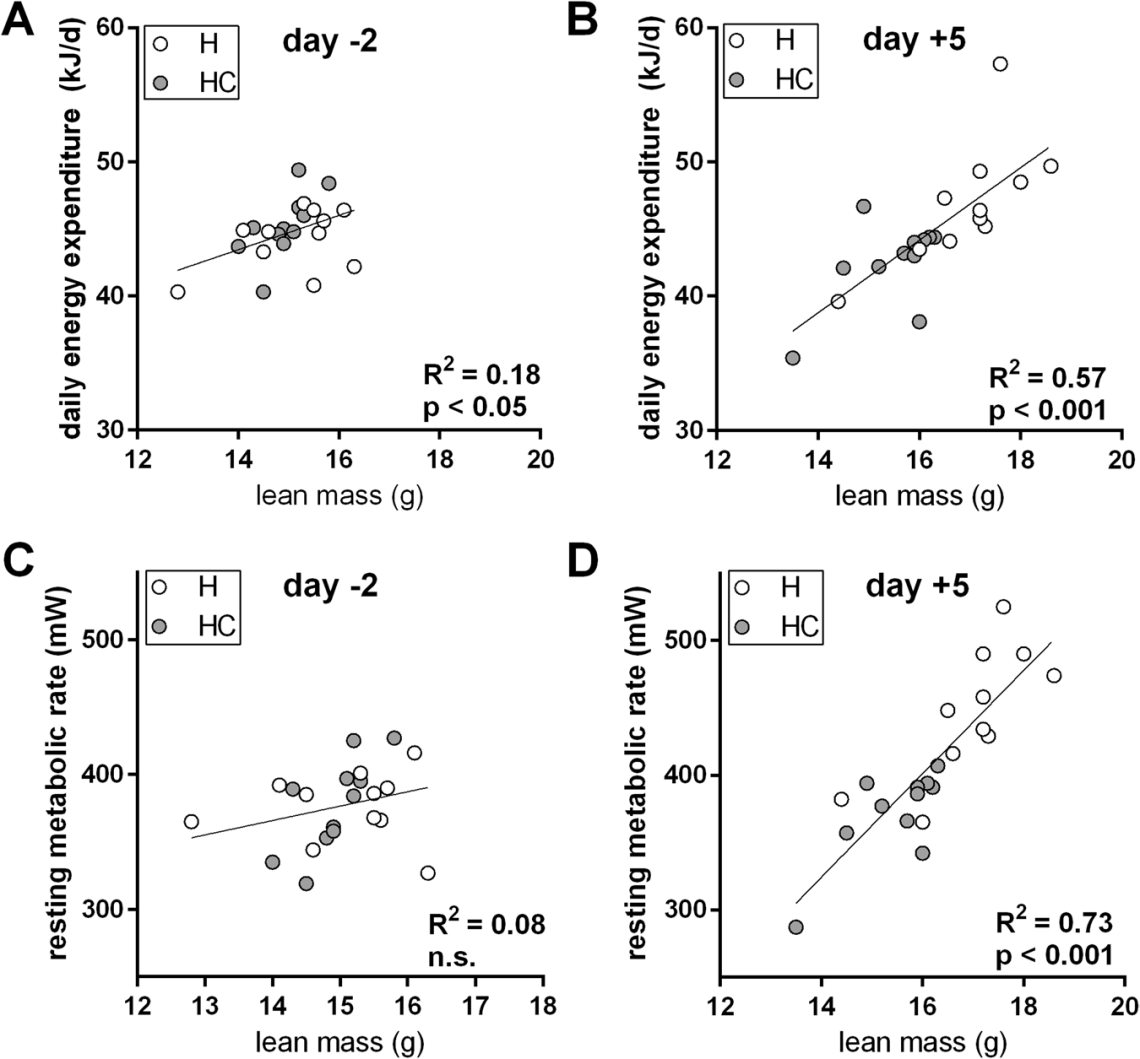
Daily energy expenditure (DEE) and resting metabolic rate (RMR) in C57BL/6J mice fed a high fat diet supplemented with cholate (HC, grey fill) or not (H, white fill). (**A**) DEE as a function of lean mass before start of the feeding trial. Grey and white color identifies future assignment to diet groups formed the next day. (**B**) DEE as a function of lean mass five days after diet change. (**C**) RMR as a function of lean mass before start of the feeding trial. Grey and white color identifies future assignment to diet groups formed the next day. (**D**) DEE as a function of lean mass five days after diet change. Goodness of fit (R^2^) and significance of a common regression is provided per panel. Separate regressions were statistically rejected (details in material and methods).

Energy expenditure is dependent on body mass and composition with a dominant contribution of lean mass. We adjusted daily energy expenditure (DEE) before and at the end of our feeding trial for individual lean mass on these days (Fig. 7A,B). At all times, mice displayed a DEE appropriate for their individual lean mass regardless of dietary bile acid supplementation. Alternative adjustments for total body mass as well as for differently weighted lean and fat mass as proposed by others^19^ all corroborated the key observation, i.e. a linear regression across both diet groups without any indication for increased energy expenditure caused by dietary bile acid supplementation (Suppl. Figs S4A,B, S5A,B). Adjusted to any one of these cause variables, DEE remained unchanged by cholate supplementation (Suppl. Fig. S6A–D).

The difference in unadjusted energy expenditure seemed larger during the daytime (Fig. 6). We therefore analyzed resting metabolic rate (RMR) to detect differences in energy expenditure that may be masked by activity otherwise. Adjustment of RMR to lean mass, body mass or body composition, however, did not reveal any difference caused by cholate feeding (Figs. 7C,D, Fig. S4C and D, Suppl. Fig. S5C and D). Adjusted to any one of these cause variables, RMR remained unchanged by cholate supplementation (Suppl. Fig. S6E–H).

In summary, cholate supplementation led to protection from body mass gain associated with higher lipid oxidation at rest. A delayed onset of feeding after waking implies food aversion that may be causally linked to different body mass trajectories. Neither changes in DEE nor in RMR contributed to this phenomenon, both of which were identical after adjustment for differences in body mass and composition. Energy expenditure did not increase in response to dietary cholate supplementation.

### Bile acid mediated protection from diet-induced, increased body mass gain is independent of uncoupling protein 1

Bile acid conferred resistance to diet-induced body mass gain in C57BL/6J mice has been hypothesized to be mediated by an increased energy expenditure caused by UCP1-dependent, non-shivering thermogenesis in brown adipose tissue (BAT)^1–3^. In our study, we observed no evidence for increased energy expenditure as a consequence of cholate supplementation. Furthermore, bile acids failed to activate UCP1-dependent thermogenesis in cultured brown adipocytes (Suppl. Fig. 2B). Nevertheless, we conducted a four-week feeding trial with mice genetically devoid of UCP1 (UCP1-KO) to clarify any contribution of brown fat thermogenesis. This experiment was conducted at thermoneutrality to eliminate superimposing effects of cold-induced thermogenesis.

**Figure 8:**
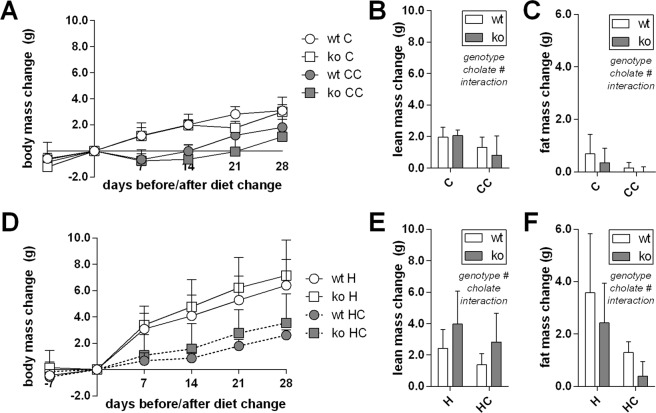
Dietary bile acid supplementation in two mouse strains, C57BL/6J wildtype (circles) and Ucp1-KO (squares), fed control (panels A–C) or high fat diet (panels D–F) (C and H, white fill) and the respective versions supplemented with cholate (CC and HC, grey fill). (**A**) Body mass change trajectories along four weeks of feeding a control diet. Cholate supplementation decreased body mass accumulation without a difference between genotypes (LME). (**B**) Lean mass after completion of the feeding trial shown in panel A. (**C**) Fat mass after completion of the feeding trial shown in panel A. (**D**) Body mass change trajectories along four weeks of feeding a high fat diet. Cholate supplementation decreased body mass accumulation without a difference between genotypes (LME). (**E**) Lean mass after completion of the feeding trial shown in panel D. (**F**) Fat mass after completion of the feeding trial shown in panel D. All data are mean values ± SD; n = 6–7 (**A**–**C**) and 5–6 (**D**–**F**); results of 2-way-ANOVA in italics (**B**,**C**,**E**,**F**) with ^#^significant effect; LME = tested by linear mixed effects model fit, see materials and methods.

High fat diet feeding led to increased body mass and fat mass accumulation as compared to mice fed a control diet (Fig. 8A–F). This difference was already detectable after one week. Supplementation of cholate attenuated body mass and fat mass gain. In all parameters, UCP1-KO mice were comparable to wildtype controls. If anything, UCP1-KO mice displayed a trend to less fat mass accumulation on a high fat diet supplemented with cholate than wildtype mice and were thus even more protected from diet induced body mass gain (Fig. 8F). It follows that the protective effect exerted by cholate supplementation is not mediated by UCP1-dependent brown fat thermogenesis as suggested previously^1–3^, in line with the concomitant absence of increased energy expenditure.

## Discussion

Dietary supplementation of bile acids leads to protection from diet-induced, increased body mass gain in C57BL/6J mice. Over the past few years, the underlying chain of events has been elaborated to include accumulation of excess bile acids in the enterohepatic organ, spillover of bile acids into systemic circulation, binding to GPBAR1 on the surface of brown adipocytes and–directly or via thyroid hormone signaling–recruitment and activation of UCP1-dependent non-shivering thermogenesis^1–3,5,7,8^. In this study, we retraced the links of this conjecture (1.) in mice of the C57BL/6J strain as studied before, (2.) a UCP1-KO mouse line on the same background and (3.) 129S6/SvEvTac mice known for their large capacity to recruit ectopic brite adipocytes^13^.

In mice of the C57BL/6J strain, dietary supplementation of cholate leads to both increased systemic plasma concentrations of bile acids and protection from diet-induced, increased body mass gain^1^. In white fat, we corroborated the moderate increase in UCP1 transcript abundance reported earlier^2,3^, an effect probably caused by direct interaction of bile acids with GPBAR1^12^. All of these consequences are completely absent in a commonly used mouse strain, 129S6/SvEvTac. This almost digital difference in the metabolic response was associated with a drastically lower amount of bile acids in the enterohepatic organ and in systemic circulation. It seems that the metabolic effects of cholate supplementation in mice of the C57BL/6J strain are directly caused by systemically circulating bile acids. It thus remains to be clarified which of the two strains is representative for other mouse strains, other commonly used animal models and humans. Disconcertingly, a large body of literature on effects of dietary cholate supplementation relies on experiments with the C57BL/6J mouse strain^1–3,5,7,8^. Past conclusions will have to be reviewed in the light of the very specific genetic background leading to exceptionally high bile acid sequestration and spillover in this strain.

In mice of the C57BL/6J strain, bile acid supplementation leads to protection from diet-induced, increased body mass gain; a phenomenon robustly reproduced multiple times in this and earlier studies^1–3^. The underlying mechanism is less clear. Bile acid supplementation has been hypothesized to exert its metabolic effects by increasing energy expenditure. The supporting studies^1,7,8^, however, measured energy expenditure for a short time only (<24 h) and normalized by division by body mass, a procedure not advisable in animals of different body mass and composition^20,21^. Two independent studies by other laboratories measuring the impact of dietary bile acid supplementation did not find an effect on energy expenditure^3,5^. In our study, we determined both daily energy expenditure and resting metabolic rate for five days. We adjusted these metabolic parameters for differences in body mass and composition. During one week after diet change, energy expenditure did not increase in response to cholate supplementation, neither in absolute terms nor adjusted, neither at rest nor as a daily budget, and neither in our hands nor in other laboratories^3,5^.

In the absence of an increased energy expenditure and without effects of bile acids on brown adipocyte respiration, the contribution of brown fat thermogenesis to an altered, short-term body mass trajectory conferred by cholate supplementation is questionable. We nevertheless conducted the appropriate experiment to investigate this possibility by including UCP1-KO mice into a feeding trial. This experiment was conducted at thermoneutrality to eliminate superimposing effects of cold-induced thermogenesis. We clearly demonstrate that UCP1-dependent non-shivering thermogenesis in brown or brite adipocytes is not required for bile acid mediated protection from diet induced, increased body mass gain during the first week of our feeding trial.

This conclusion is in disagreement with the results of a very similar experiment with the same mouse strain conducted unknowingly in parallel in L. Kozak’s laboratory^2^. Here, UCP1 seemed partially required for bile acid mediated protection from diet induced, increased body mass gain. However, even the role of UCP1 itself in body mass regulation is not fully resolved. In different studies, the identical UCP1-KO mouse strain is more^22–24^, less^25,26^ or similarly prone to diet induced, increased body mass gain (this study^2,27^), even from cohort to cohort within the same colony^28^. It is hard to conceive how a protective effect of bile acids can be reliably reproducible in C57BL/6J mice when the underlying mechanism is not. If it does not even robustly require the presence of UCP1, the stringent conclusion is that bile acid mediated protection from diet induced, increased body mass gain does not involve changes in energy expenditure caused by uncoupling in brown or brite adipocytes.

In summary, we report that protection from diet induced, increased body mass gain by dietary bile acid supplementation is mouse strain specific, does not involve increased energy expenditure and does not require UCP1-dependent, non-shivering thermogenesis during the first week of intervention.

## Methods

### Animals and Housing

All animal experimentation was conducted according to the German Animal Welfare Act and previously approved by the relevant authority (Government of Upper Bavaria, file no. 55.2-1-54-2532-15-14). We employed male mice of the C57BL/6J and 129S6/SvEvTac inbred strains bred in our specified pathogen-free facility at 50–60% relative humidity, 22 °C ± 1 °C and a 12-hour light/dark cycle. All mice received standard rodent chow diet (V1124-300, Ssniff Spezialdiäten GmbH) prior to experiments. Uncoupling protein 1 (Ucp1) knockout (KO) mice on C57BL/6J background were generated by L. Kozak and coworkers^29,30^ and founder mice were kindly provided to establish our colony. Wildtype (WT) and Ucp1-KO mice obtained from heterozygous breeding were mated in homozygous WT/WT and KO/KO breeding pairs. These pairs were kept at 30 °C inside a climate cabinet (HPP749, Memmert) where F1-offspring was born, raised and subjected to experiments.

### Dietary Bile Acid Supplementation Experiments

Data were collected in three independent experiments characterized by the administration of four diets. Mice received a control (C, S5745-E702) or a high fat (H, S5745-E712) diet providing 13% or 48% of energy from fat, respectively. To investigate the effect of dietary bile acid administration, these diets were supplemented with 0.5% (w/w) sodium cholate at the expense of cornstarch to generate C + cholate (CC, S5745-706) and H + cholate (HC, S5745-716) diets. All diets were obtained from Ssniff Spezialdiäten GmbH and fed *ad libitum*.

In experiment 1, individually housed C57BL/6J and 129S6/SvEvTac mice were switched to experimental diets at the age of 6–7 weeks for four weeks. Body composition (absolute lean and fat mass) was determined at the end of the dietary intervention by nuclear magnetic resonance (mq 7.5, Bruker). Food intake was determined from manually weighed food mass to calculate daily cholic acid intake of bile acid-supplemented mice. Feces were collected during the fourth week of feeding to determine fecal bile acid excretion.

In experiment 2, C57BL/6J mice were provided control diet starting at the age of 4–5 weeks. After 12 days (day −2), mice were subjected to body mass and composition measurements, single-caged and transferred to the indirect calorimetry setup. Two days later (day 0), mice were switched to high fat diet with or without cholate. Body mass and composition measurements were repeated after five more days within the indirect calorimetry setup (day +5).

In experiment 3, WT und Ucp1-KO mice received control diet at 6–7 weeks of age. One week later, mice were switched to experimental diets and fed for four weeks. Body composition changes were assessed by nuclear magnetic resonance measurements at the beginning and at the end of the bile acid supplementation. Experiment 3 was performed under thermoneutral conditions with mice bred and raised at thermoneutrality (30 °C).

### Indirect Calorimetry

Cages with mice were placed inside a climate cabinet (TPK 600, Feutron) at 23 °C. Energy expenditure was assessed by indirect calorimetry in an open flow respiratory system (Phenomaster, TSE Systems) within the cabinet. Air was extracted from the cages with a flow rate of 0.7 l/min over a period of 1 minute in 9-minute intervals to determine volumes of oxygen consumed, carbon dioxide produced and the respiratory exchange ratio (RER). Heat production was calculated as [mW] = (4.44 + 1.43 * RER) * oxygen consumed [ml/h]. Daily energy expenditure (DEE) was calculated as the sum of energy expenditure values during all 9-minute intervals of a given day. Resting metabolic rate (RMR) was calculated as the lowest mean heat production of four consecutive 9–minute intervals (=36 minutes).

### Cell Culture

The generation of immortalized preadipocytes from the stromal vascular fraction of inguinal white adipose tissue has been reported previously^31^. Cells were grown to confluence in culture medium comprising Dulbecco’s modified Eagle medium (4.5 g/l glucose, GE Healthcare Bio-Sciences), 20% fetal bovine serum (Life Technologies), 20 nM insulin and 1 nM T3. Adipocyte differentiation was induced by complementing the medium with 250 µM indomethacin, 500 µM isobutylmethylxanthine and 2 µg/ml dexamethasone for 24 hours after confluence. During six days of differentiation, the medium was supplemented or not (control) with one of the following compounds (each at 20 µM): rosiglitazone (Biomol), cholate, glycodeoxycholate, ursodeoxycholate, taurodeoxycholate, chenodeoxycholate, taurocholate, deoxycholate, tauroursodeoxycholate, taurochenodeoxycholate, glyocholate (all Sigma-Aldrich) or tauro-beta-muricholate (Santa Cruz). Primary brown adipocytes were isolated, cultured and differentiated from the stromal vascular fraction of interscapular BAT of male 129S6/SvEvTac mice as described previously^13^.

### Cellular respiration assay

Oxygen consumption rate of brown adipocytes differentiated for six days was measured at 37 °C (XF96 Flux Analyzer, Seahorse Bioscience) as described previously^10^. To test the acute thermogenic potential of bile acids, UCP1-mediated uncoupled respiration was determined after stimulating with various concentrations of LCA and CDCA under buffered condition with 2% essentially fatty acid free bovine serum albumin in the respiration medium. Isoproterenol stimulation served as a positive control. Oxygen consumption rates were automatically calculated. Data were exported, reconstructed and analyzed in GraphPad Prism 6.

### RNA isolation and quantitative real-time PCR

Cells or tissues were homogenized in an appropriate volume of TRIsure (Bioline) and processed according to the manufacturer’s instructions. Precipitated RNA was further purified by spin columns (SV Total RNA Isolation System, Promega). Concentrations of RNA eluates were determined spectrophotometrically (Infinite 200 Pro NanoQuant, Tecan). Synthesis of complementary DNA (cDNA) was performed with the QuantiTect Reverse Transcription kit (Qiagen) using 500 ng of RNA in a final volume of 10 µl. Quantitative real-time PCR was conducted on multiwall plates (Mastercycler ep realplex epgradient, Eppendorf or LightCycler 480, Roche) in a reaction comprising 2x SensiMix SYBR No-ROX (Bioline), 400 nM forward and reverse primers and template cDNA. Primers were produced by Eurofins Genomics and sequences were as follows: Ucp1: for: 5′-TCT CTG CCA GGA CAG TAC CC-3′, rev: 5′-AGA AGC CCA ATG ATG TTC AG-3′; Cidea: for: 5′-TGC TCT TCT GTA TCG CCC AGT-3′, rev: 5′-GCC GTG TTA AGG AAT CTG CTG-3′; Cox7a1: for: 5′-CCG ACA ATG ACC TCC CAG TA-3′, rev: 5′-TGT TTG TCC AAG TCC TCC AA-3′; Otop1: for: 5′-ACT AGG ACC CCG TCG AAT CT-3′, rev: 5′-ACC ATG CTC TAC GTG CTG TG-3′; Hsp90: for: 5′-AGG AGG GTC AAG GAA GTG GT-3′, rev: 5′-TTT TTC TTG TCT TTG CCG CT-3′; Actb: for: 5′-AGA GGG AAA TCG TGC GTG AC-3′, rev: 5′-CAA TAG TGA TGA CCT GGC CGT-3′; Hprt: for: 5′-CAG GCC AGA CTT TGT TGG AT-3′, rev: 5′-TTG CGC TCA TCT TAG GCT TT-3′; Cyp7a1: for: 5′-CCG GAC AGC TAA GGA GGA CTT C-3′; rev: 5′-TGT AGC TCC TGA TCC AAA GGG C-3′; Slc10a2: for: 5′-GTG GTG ATT GCC TCC AAC ATC G-3′, rev: 5′-AGG GTG GGA GGT GTG AGT ATC T-3′; Slc51a: for: 5′-GCT TGC TCA CCT CCC TAC TCT T-3′, rev: 5′-TAT GTC TTC CAG GGT CCA AGC C-3′; Slc51b: for: 5′-TCC TGG TGG TCA TGA CAA GCA T-3′, rev: 5′-CGT TAT GGG GCG TTA TGG GGT A-3′. Transcript abundance was quantified relative to a standard curve of pooled cDNA in a 2^8^-fold dilution series. Expression levels of target genes were normalized to the mean of heat shock protein 90 (Hsp90) and beta-actin (Actb) abundance (in adipose tissues) or to Hprt (in liver and ileum).

### Histology

Dissected tissues were fixed in phosphate buffered saline with 4% paraformaldehyde and 0.0024% picric acid for one week. Following dehydration, 5 µM sections of paraffin-embedded tissues were mounted on object slides and subjected to hematoxylin-eosin-staining or immunohistochemistry. For immunohistochemical analyses, sections were cleared from paraffin, and subjected to sodium citrate-mediated epitope retrieval at 90 °C for 30 minutes and further incubated in 3% H_2_O_2_ for 10 minutes to saturate endogenous peroxidases. Sections were blocked in phosphate buffered saline with 2.5% normal goat serum for 1 hour prior to the overnight-incubation of the primary antibody at 4 °C (1:500 rabbit anti-UCP1 (Abcam, ab10983) in phosphate buffered saline comprising 0.1% Tween-20 and 0.25% normal goat serum). Sections were incubated with horseradish peroxidase-coupled secondary antibody (1:200, goat anti-rabbit, Abcam, ab97051) for 1 hour at room temperature, rinsed thrice with phosphate buffered saline containing 0.1% Tween-20, and incubated 2 minutes with diaminobenzidine solution (DAB Enhanced Liquid Substrate System, Sigma-Aldrich). Mounted sections were inspected with a bright field microscope using similar adjustments for all slides.

### Bile acid determination

Bile acids were extracted from plasma (10 µl), enterohepatic organs (40 mg of a powder generated from small intestine, large intestine, caecum, liver, gall bladder and pancreas on liquid nitrogen) and grinded feces (20 mg). Plasma was mixed with an equal volume of a bile acid mix used as internal standard. The biological materials were mixed with an appropriate volume of methanol and centrifuged with 12,000 rpm for 10 minutes at 4 °C. Supernatants were dried at 40 °C in a vacuum concentrator (SPD111V SpeedVac, Thermo Savant) and resuspended in a mixture of methanol and water. The fecal and enterohepatic resuspensions were supplemented with stable isotope labelled standards prior to the quantification of bile acids with a mass spectrometer (QTrap 5500, Sciex) operating in positive mode. Mass spectra were recorded using the multiple reaction-monitoring mode. Signal acquisition and quantification were performed using the Sciex Analyst and Multiquant software, respectively.

### Statistical analyses

Body mass development of mice fed different diets was compared by linear mixed effect model fits with the fixed effects ‘time’, ‘diet’ and ‘cholate supplementation’ (experiment 1) or ‘time’, ‘genotype’ and ‘cholate supplementation’ (experiment 3), all relevant interaction terms and ‘individual animal’ as random effect. Final parameters of experiments 1 and 3 were evaluated with 2-way-ANOVAs with the factors ‘diet’, ‘cholate supplementation’ and their interaction. To identify the source of interaction we performed appropriate post-hoc tests (t-test; Sidak’s multiple testing).

The interrelation of daily energy expenditure or resting metabolic rate with body mass, lean mass or body composition was analyzed by regression analysis (least square fit for a straight line). We first tested whether the regressions within the two groups ‘exposed to cholate supplemented diet’ and ‘exposed to non-supplemented diet’ were significantly different (extra sum-of-squares F test) and rejected this hypothesis in favor of a common regression when it failed to reach significance or had a goodness of fit with less explanatory power than a horizontal line (negative R^2^).

Statistical analyses were performed with GraphPad Prism 6 or Matlab R2016b (linear mixed effect model fits).

## Supplementary information

Supplementary figures

## Data Availability

The datasets generated and analyzed during this study are available from the corresponding author on reasonable request.
