# Supplementary material for: Bile acid supplementation decreases body mass gain in C57BL/6J but not 129S6/SvEvTac mice without increasing energy expenditure
Source: Sci Rep. 2019 Jan 15;9:131. doi: 10.1038/s41598-018-37464-z (PMC6333827; doi:10.1038/s41598-018-37464-z)
Supplement: Supplementary file 1 — Supplementary figures [file 41598_2018_37464_MOESM1_ESM.pdf]

Bile acid supplementation decreases body mass gain in C57BL/6J but not 129S6/SvEvTac mice without increasing energy expenditure

Tobias Fromme*,#,[1,2], Kristina Hüttinger*[1,2], Stefanie Maurer[1,2], Yongguo Li[1,2], Thomas Gantert[1,2], Jarlei Fiamoncini[4], Hannelore Daniel[4], Sören Westphal[5], Martin Klingenspor[1,2,3]

[1]: Chair of Molecular Nutritional Medicine, TUM School of Life Sciences, Technical University of Munich, Freising, Germany
[2]: EKFZ - Else Kröner-Fresenius Center for Nutritional Medicine, Technical University of Munich, Freising, Germany
[3]: ZIEL - Institute for Food & Health, Technical University of Munich, Freising, Germany.
[4]: Molecular Nutrition Unit, Technical University of Munich, Freising, Germany
[5]: Department of Internal Medicine II, University of Ulm, Ulm, Germany

* these authors contributed equally to this work

# corresponding author:
Dr. Tobias Fromme
Technical University of Munich
Chair of Molecular Nutritional Medicine
Gregor-Mendel-Str. 2
85354 Freising, Germany
Tel. +49.8161.71.3850
fromme@tum.de

Supplemental Figure S1

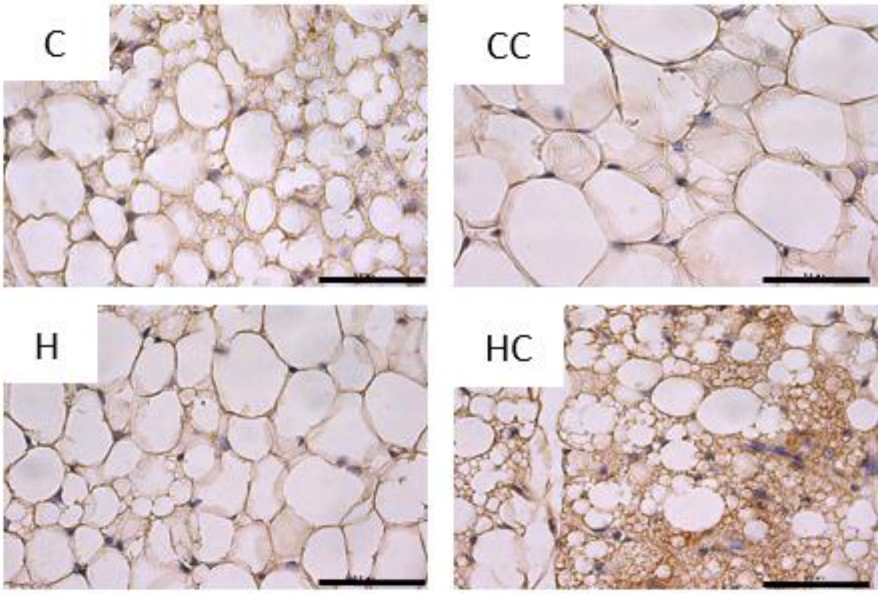

Supplemental Figure S1 – Immuno-histological detection of uncoupling protein 1 on representative iBAT sections from the four diet groups of C57BL/6J mice, scale bar 50 µm.

Supplemental Figure S2

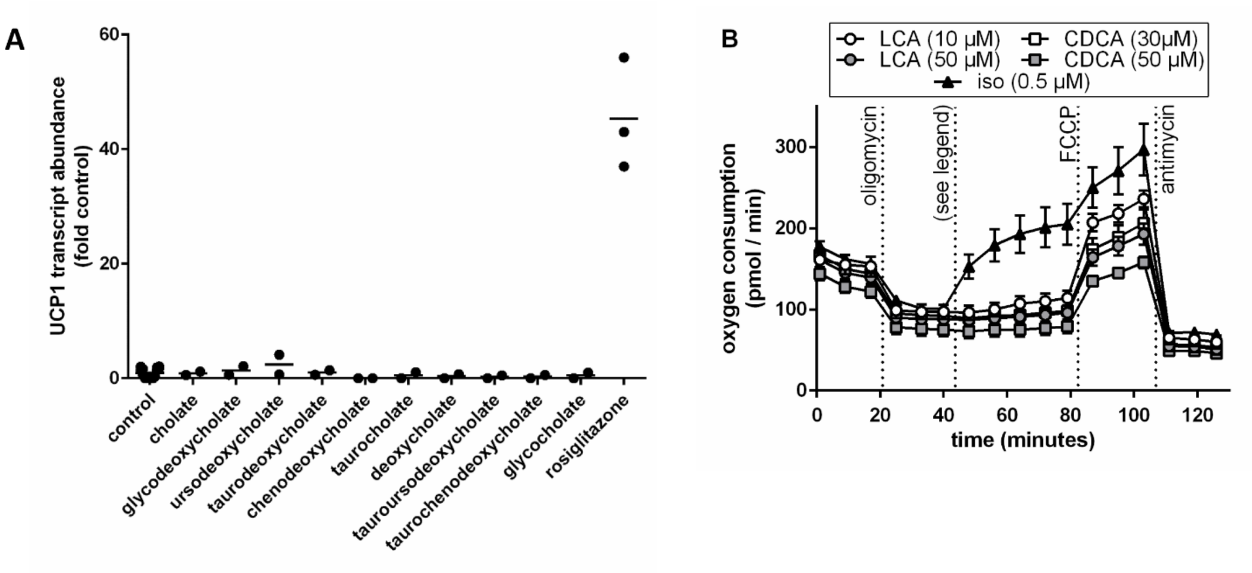

Supplemental Figure S2 – Bile acid effects in cultured cells. **A -** Transcript abundance of uncoupling protein 1 (UCP1) in fully differentiated, immortalized adipocytes after 6 days of treatment with different bile acids or the positive control compound rosiglitazone. None of the bile acids tested exhibits a potential to recruit brite adipocytes in this treatment regime. **B –** Oxygen consumption of primary brown adipocytes treated with isoproterenol (iso), litocholate (LCA) or chenodeoxycholate (CDCA) after inhibition of ATP synthesis (oligomycin). Carbonyl cyanide-4-(trifluoromethoxy)phenylhydrazone (FCCP) fully uncoupled cells and inhibition of complex III by antimycin revealed non-mitochondrial oxygen consumption. None of the bile acids tested thus exhibited a potential for the generation of brite or activation of brown adipocytes in there treatment regimes.

Supplemental Figure S3

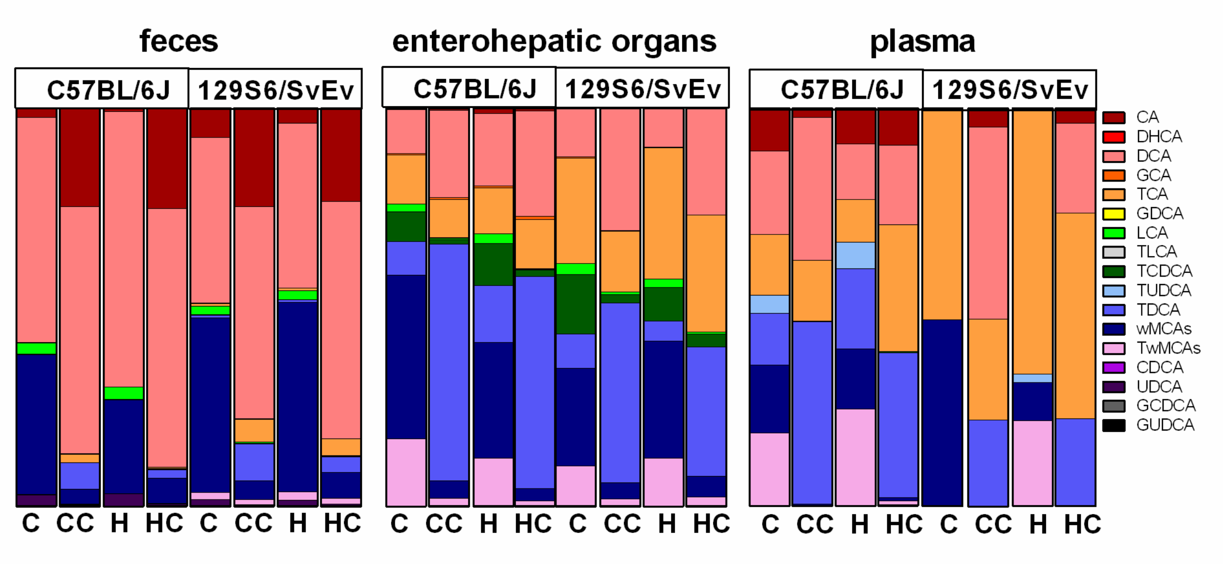

Supplemental Figure S3 – Composition of bile acid pools in feces, enterohepatic organs and plasma of two mouse strains, C57BL/6J and 129S6/SvEvTac, fed control or high fat diet (C and H) alternatively supplemented with cholate (CC and HC). Every bar section represents the median molar concentration as a fraction of total pool size (n= 4-7). CA – cholate, DCA – deoxycholate, TCA – taurocholate, GDCA – glycodeoxycholate, LCA – lithocholate, TCDCA – taurochenodeoxycholate , TDCA – taurodeoxycholate , wMCAs – omega muricholates, CDCA – chenodeoxycholate, UDCA – ursodeoxycholate, TUDCA – tauroursodeoxycholate, DHCA – dehydrocholate, GCA – glycocholate, TLCA – taurolithocholate , TwMCAs – tauro-omega-muricholates, GCDCA – glycochenodeoxycholate, GUDCA – glycoursodeoxycholate.

Supplemental Figure S4

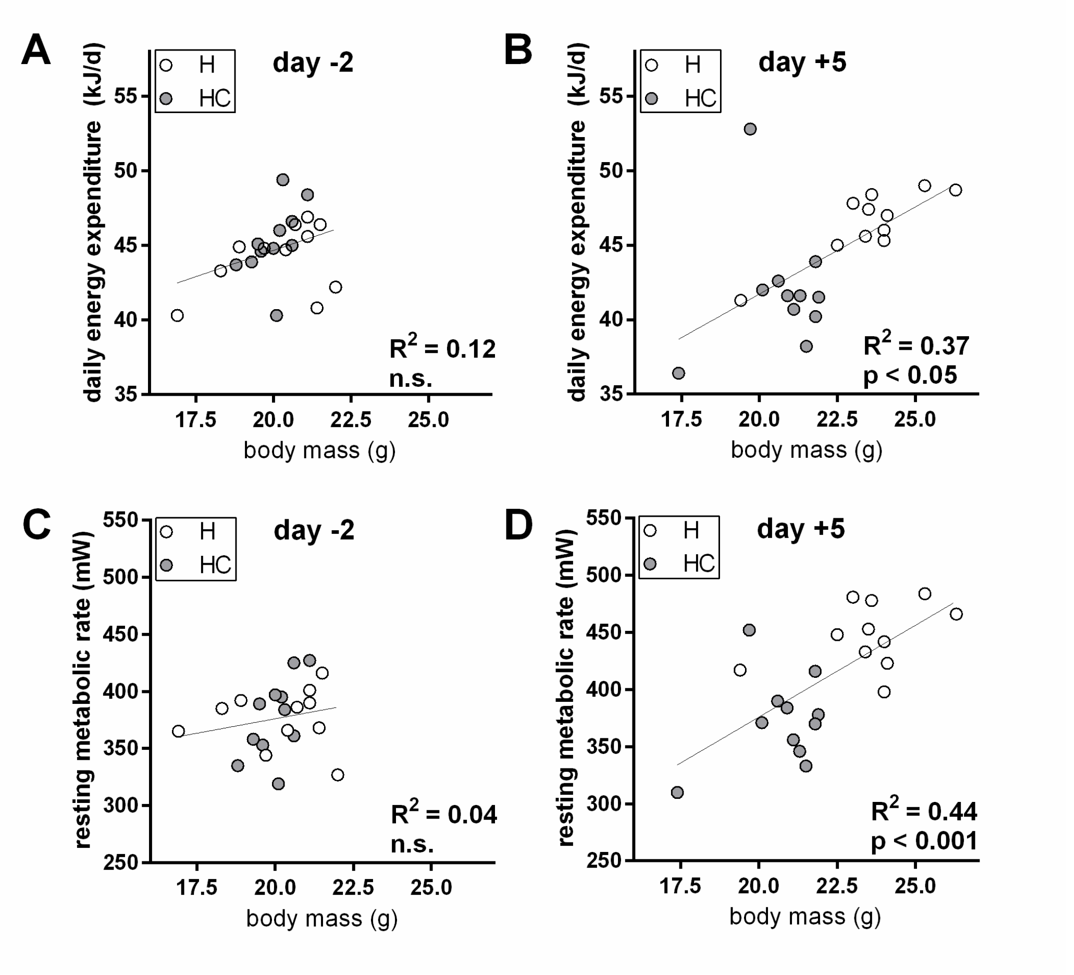

Supplemental Figure S4 – Daily energy expenditure (DEE) and resting metabolic rate (RMR) in mice fed a high fat diet supplemented with cholate (HC, grey fill) or not (H, white fill). **A** – DEE as a function of body mass before start of the feeding trial. Grey and white color identifies future assignment to diet groups formed the next day. **B** – DEE as a function of body mass five days after diet change. **C** – RMR as a function of body mass before start of the feeding trial. Grey and white color identifies future assignment to diet groups formed the next day. **D** – DEE as a function of body mass five days after diet change. Goodness of fit (R^2) and significance of a common regression is provided per panel. Separate regressions were statistically rejected (details in material and methods).

Supplemental Figure S5

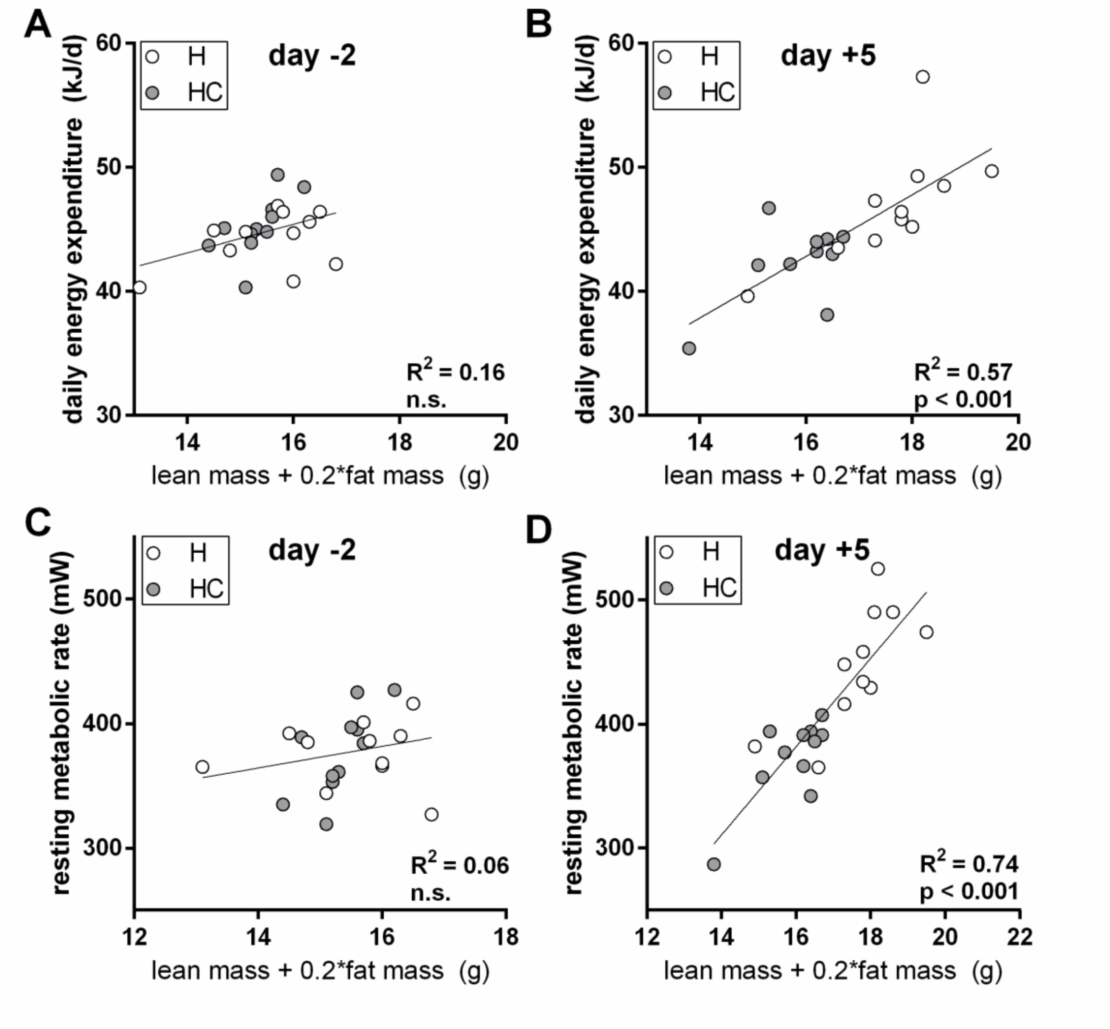

Supplemental Figure S5 – Daily energy expenditure (DEE) and resting metabolic rate (RMR) in mice fed a high fat diet supplemented with cholate (HC, grey fill) or not (H, white fill). Parameter are depicted as a function of body composition calculated as ‘lean mass + 0.2* fat mass‘. **A –** DEE as a function of body composition before start of the feeding trial. Grey and white color identifies future assignment to diet groups formed the next day. **B –** DEE as a function of body composition five days after diet change. **C –** RMR as a function of body composition before start of the feeding trial. Grey and white color identifies future assignment to diet groups formed the next day. **D –** DEE as a function of body composition five days after diet change. Goodness of fit (R^2) and significance of a common regression is provided per panel. Separate regressions were statistically rejected (details in material and methods).

Supplemental Figure S6

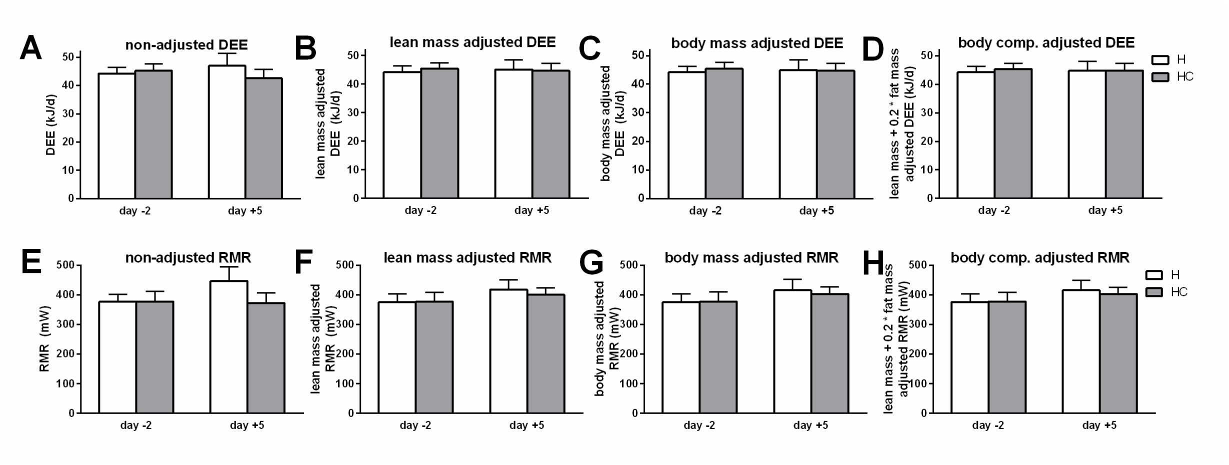

Supplemental Figure S6 – Neither daily energy expenditure (DEE) nor resting metabolic rate (RMR) are increased in mice fed a high fat diet supplemented with cholate (HC, grey fill) as compared to unsupplemented high fat diet (H, white fill). **A** – Unadjusted DEE. **B** – Lean mass adjusted DEE. **C** – Body mass adjusted DEE. **D** – Body composition (lean mass + 0.2 fat mass) adjusted DEE. **E** – Unadjusted RMR. **F** – Lean mass adjusted RMR. **G** – Body mass adjusted RMR. **H** – Body composition (lean mass + 0.2 fat mass) adjusted RMR. In all panels, bar color on day -2 (all mice on control diet) refers to future group assignment.

Supplemental Figure S7

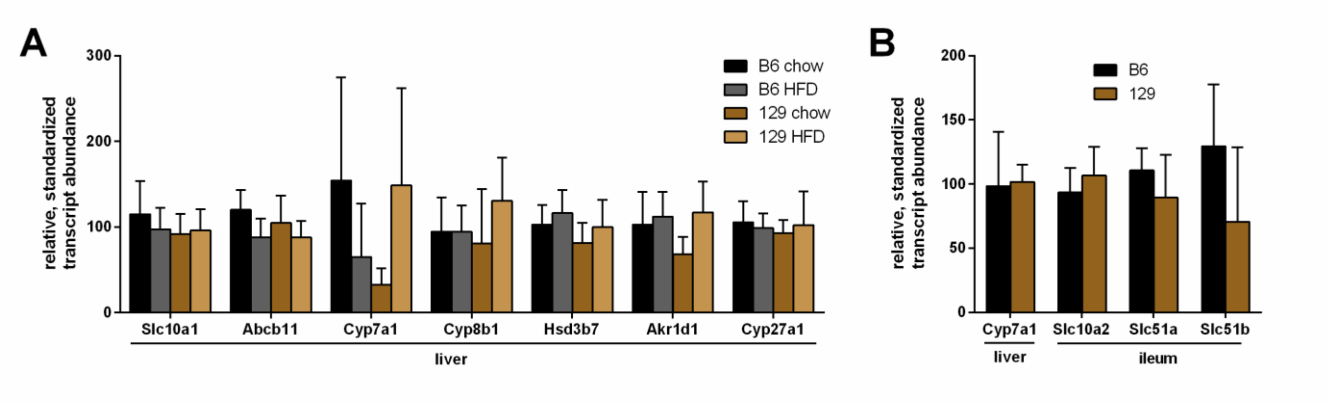

Supplemental Figure S7 – Transcript abundance of bile acid handling genes is not different between mouse strains C57Bl/6J (B6) and 129S6/SvEv (129). **A –** Relative transcript abundance of bile acid transport and metabolic genes in the liver of chow or high fat diet (HFD) fed mice of either strain. Data taken from publicly available dataset (GEO accession GSE45684). **B –** Quantification by qPCR of transcript abundance of selected genes in our own mice of the same strains, normalized to Hprt. Neither Cyp7a1 in liver, nor bile acid transporters in ileum are differentially expressed.
